# Supplementary material for: Nanopore adaptive sampling effectively enriches bacterial plasmids
Source: mSystems. 2024 Feb 20;9(3):e00945-23. doi: 10.1128/msystems.00945-23 (PMC10949517; doi:10.1128/msystems.00945-23)
Supplement: Supplemental Material — Figures S1-S9 and Table S1. [file msystems.00945-23-s0001.pdf]

SUPPLEMENTARY MATERIAL

for

Nanopore adaptive sampling effectively enriches bacterial plasmids

Jens-Uwe Ulrich [1,2,*], Lennard Epping [3], Tanja Pilz [3], Birgit Walther [4], Kerstin Stingl [5], Torsten Semmler [3] and Bernhard Y. Renard[1]

1. Hasso Plattner Institute, Digital Engineering Faculty, University of Potsdam, 14482 Potsdam, Germany
2. Department of Mathematics and Computer Science, Free University of Berlin, 14195 Berlin, Germany
3. Genome Sequencing and Genomic Epidemiology, Robert Koch Institute, 13353 Berlin, Germany
4. Advanced Light and Electron Microscopy, Robert Koch Institute, 13353 Berlin, Germany
5. National Reference Laboratory for Campylobacter, Department of Biological Safety, German Federal Institute for Risk Assessment (BfR), 12277 Berlin, Germany

* Correspondence: jens-uwe.ulrich@hpi.de , bernhard.renard@hpi.de

Investigation of the sequencing runs

This section will provide an overview of the general sequencing run and sample metrics of the control regions to assess the quality of the four sequencing runs.
First, we investigated the quality of the four sequencing runs by looking at the number of active sequencing pores, read lengths and mean Phred quality scores of reads from the control regions of the sequencing runs. First, we see that flow cell ReadBouncer1 has the highest number of active sequencing pores (1,153) at the start of the run, while the other three flow cells have between 557 and 718 active pores (Figure S1 (a)). The fewer active pores can be explained using expired flow cells for these three sequencing runs. This also shows that the number of active sequencing pores on expired flow cells is generally below the minimum number of active pores covered by the manufacturer's warranty of 800 pores.
Regarding read lengths of sequencing data after base calling from the control regions, we can only compare ReadBouncer1 with MinKNOW1 and ReadBouncer2 with MinKNOW2. For ReadBouncer1 and MinKNOW1, both having the same *Campylobacter* samples sequenced, we see a larger proportion of longer reads above 10,000 base pairs for ReadBouncer1 (Figure S1 (b)). To investigate that difference, we looked at per-sample read length metrics and distributions, which are provided in Table S1 and Figure S1 (c). Here, we recognize that the larger proportion of reads longer than 10kb in ReadBouncer1 is mainly caused by the *Campylobacter jejuni* sample. Our results also show a large difference in mean and median read lengths and the standard deviation for the two *Campylobacter* samples. Here, the application of the MagAttract HMW Genomic Extraction Kit (Qiagen), which was used for internal validation, results in larger read lengths for the *Campylobacter jejuni* samples. We also see a difference between the read lengths of the same sample from the two *Campylobacter* sequencing runs (ReadBouncer1 and MinKNOW1), particularly for *Campylobacter coli*. However, there is no trend that read lengths on expired flow cells are generally shorter because the read lengths for *Campylobacter coli* are longer on the expired flow cell MinKNOW1 when compared to ReadBouncer1.
To further assess and compare the quality of the four sequencing runs, we look at the quality from the control regions of the sequencing runs. The contour plots in Figure S6 show that for all four sequencing runs, a large proportion of reads has a mean Phred quality between 12 and 15. Performing a Mann-Whitney U-Test revealed statistically significant differences in Phred quality scores between the different sequencing runs, but the effect sizes were small with regard to Cohen's classification ($r < 0.2$).
One of the major aspects of our study is the investigation of the impact adaptive sampling has on expired nanopore flow cells. Therefore, we first compared the average read quality scores from reads sequenced on control regions (Figure S6) with those sequenced on adaptive sampling regions (Figure S7). Although we see a statistically significant difference between adaptive sampling and control regions (Mann-Whitney U Test $p < 0.05$) for all 4 experiments, the effect sizes are very small with regard to Cohen's classification ($r < 0.1$). This comparison shows no significant loss in read quality when applying adaptive sampling to expired flow cells. In a second step, we checked whether adaptive sampling leads to faster pore exhaustion on expired flow cells by investigating the effect of adaptive sampling on the number of active sequencing channels and yield in sequenced Mbases per hour (see Figure S8). Although we consistently observe more active sequencing channels in control regions, our linear regression analysis did not reveal systematically faster degradation of pores in adaptive sampling regions. We could also not detect bigger systematic differences in active sequencing channels on expired flow cells when compared to the fresh flow cell ReadBouncer1.

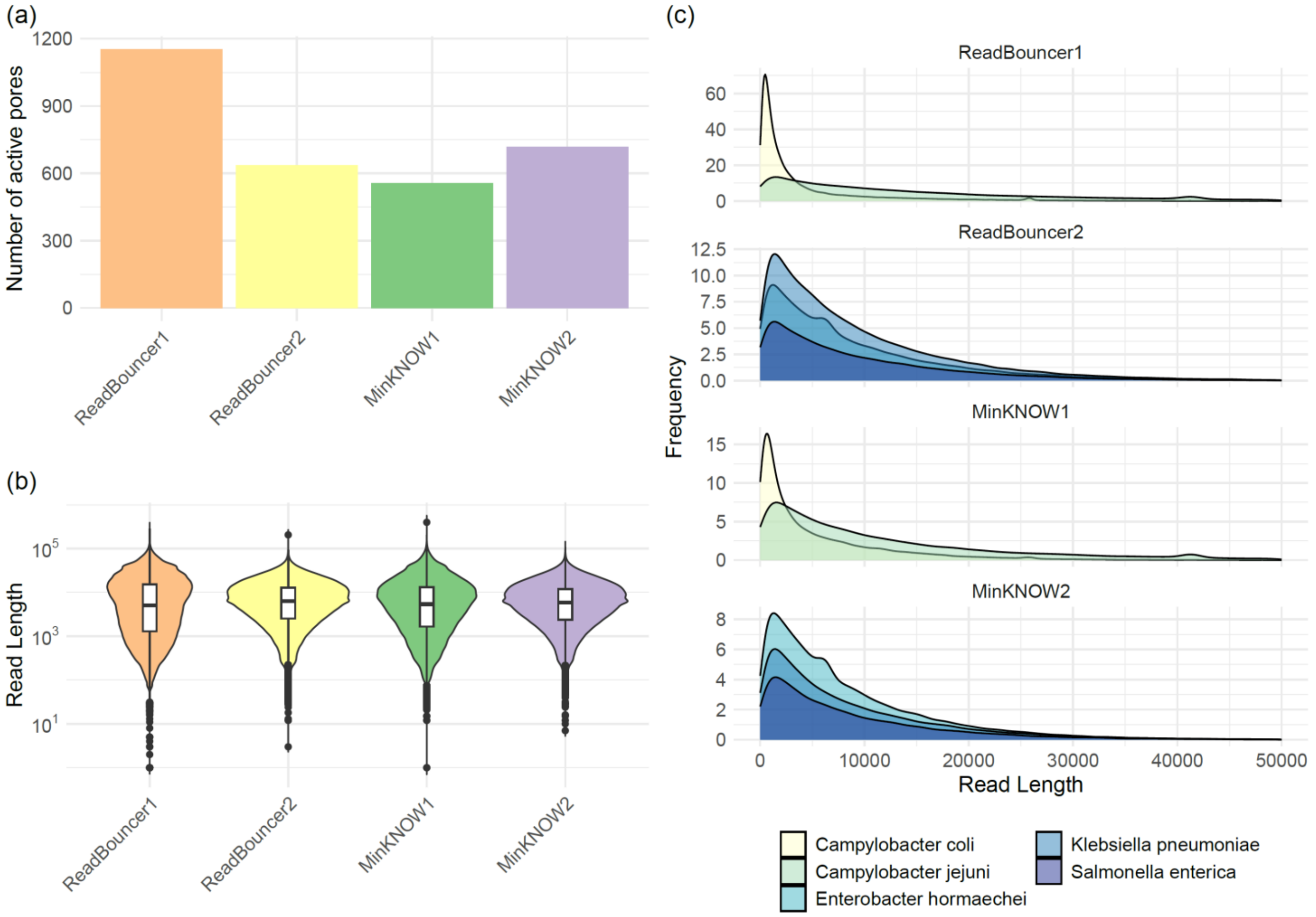

Supplementary Figure S1. Evaluation of control regions from the first four sequencing runs. (a) Active pore count measured at the start of each sequencing run. ReadBouncer1 has the highest number of active pores because it was the only flow cell that was not expired. **(b)** Violin plots (log scale) of read length distributions. Box plots for read lengths are included within the violin plots. The line splitting the box represents the median read length. The lower edge of the box represents the lower quartile and the upper edge represents the upper quartile of the read length distributions.. **(c)** Read length distributions for each species from control regions of the four sequencing runs.

Supplementary Table S1. Overview of read length metrics of the five bacterial isolates. The metrics were computed from reads sequenced on the control side of the flow cells where no adaptive sampling was applied.

Reference	Flow cell ID	Mean Length	Median Length	Std. Deviation
Campylobacter jejuni	ReadBouncer1	16,525.65	10,950	17,083.76
	MinKNOW1	13,360.65	8,192	15,072.50
Campylobacter coli	ReadBouncer1	4,375.37	1,580	6,952.86
	MinKNOW1	5,536.69	2,736	6,894.11
Salmonella enterica	ReadBouncer2	9,304.79	6,455	9,131.90
	MinKNOW2	8,679.22	5,952	8,629.98
Enterobacter hormaechei	ReadBouncer2	8,909.21	6,239	8,845.49
	MinKNOW2	8,309.64	8,861	8,192.69
Klebsiella pneumoniae	ReadBouncer2	9,379.93	6,512	9,180.22
	MinKNOW2	8,842.23	5,963	8,928.63

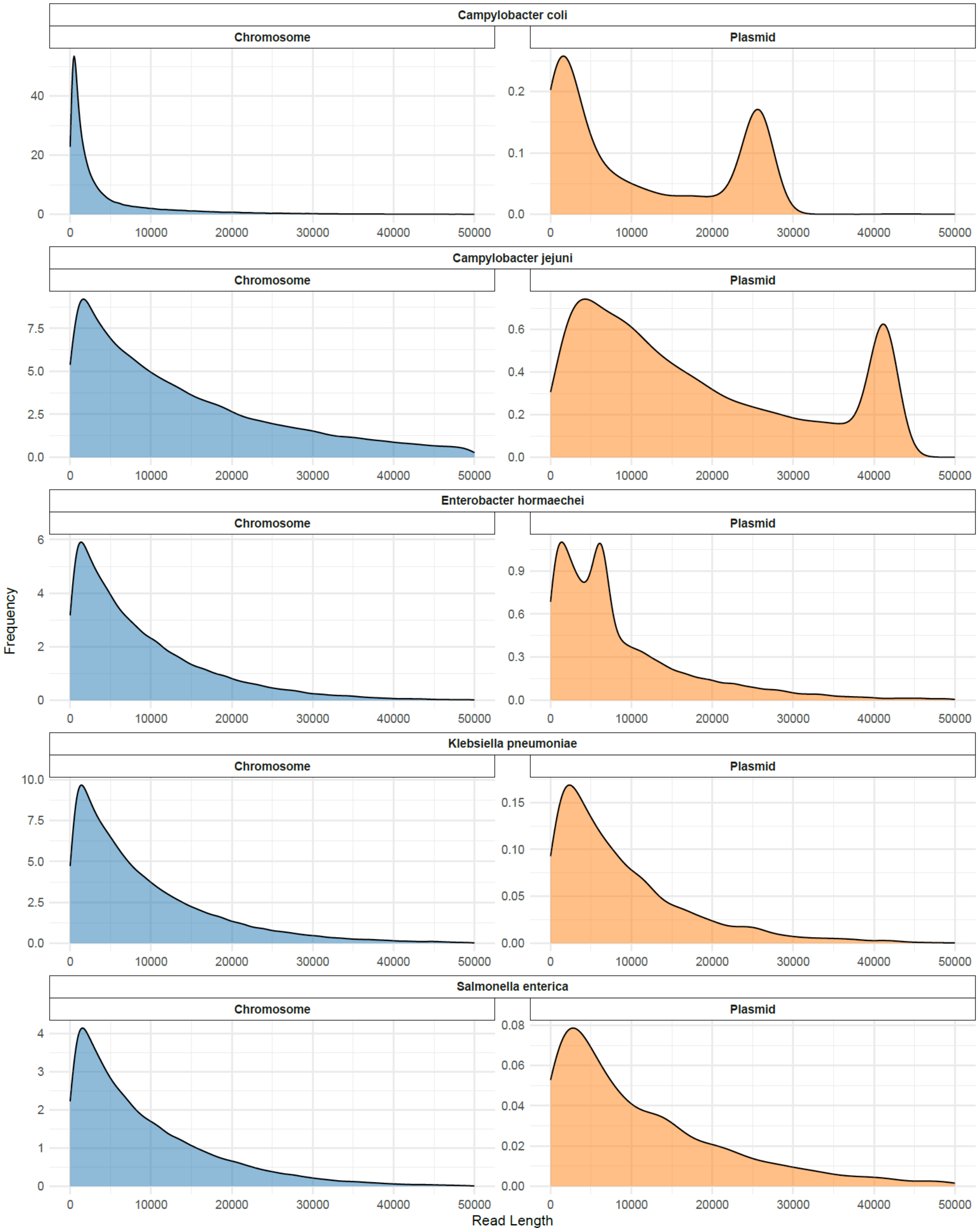

Supplementary Figure S2. Read length histograms of control regions from flow cells ReadBouncer1 and ReadBouncer2. Separate read length histograms of chromosomal and plasmid reads are shown for each of the five bacterial isolates.

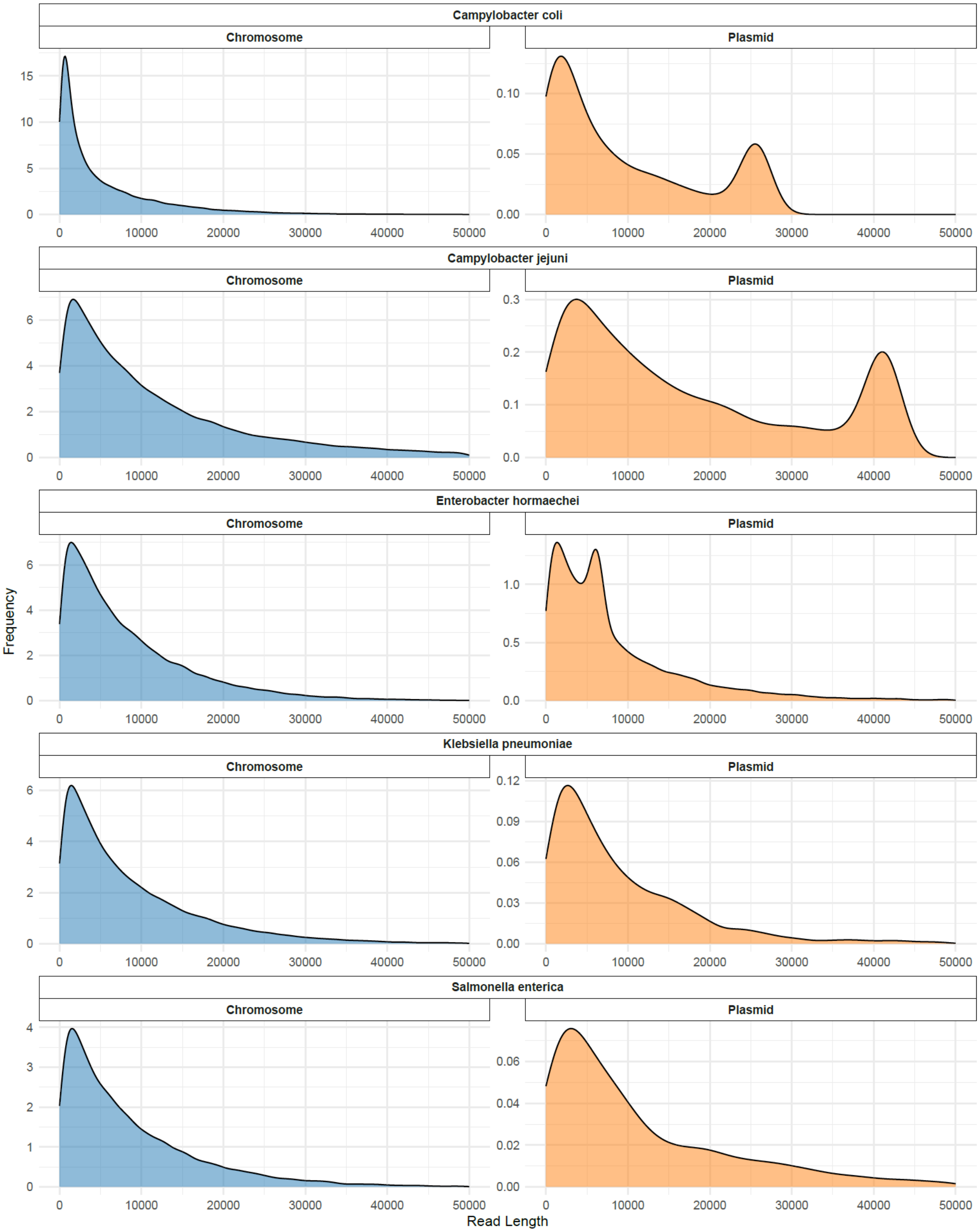

Supplementary Figure S3. Read length histograms of control regions from flow cells MinKNOW1 and MinKNOW2. Separate read length histograms of chromosomal and plasmid reads are shown for each of the five bacterial isolates.

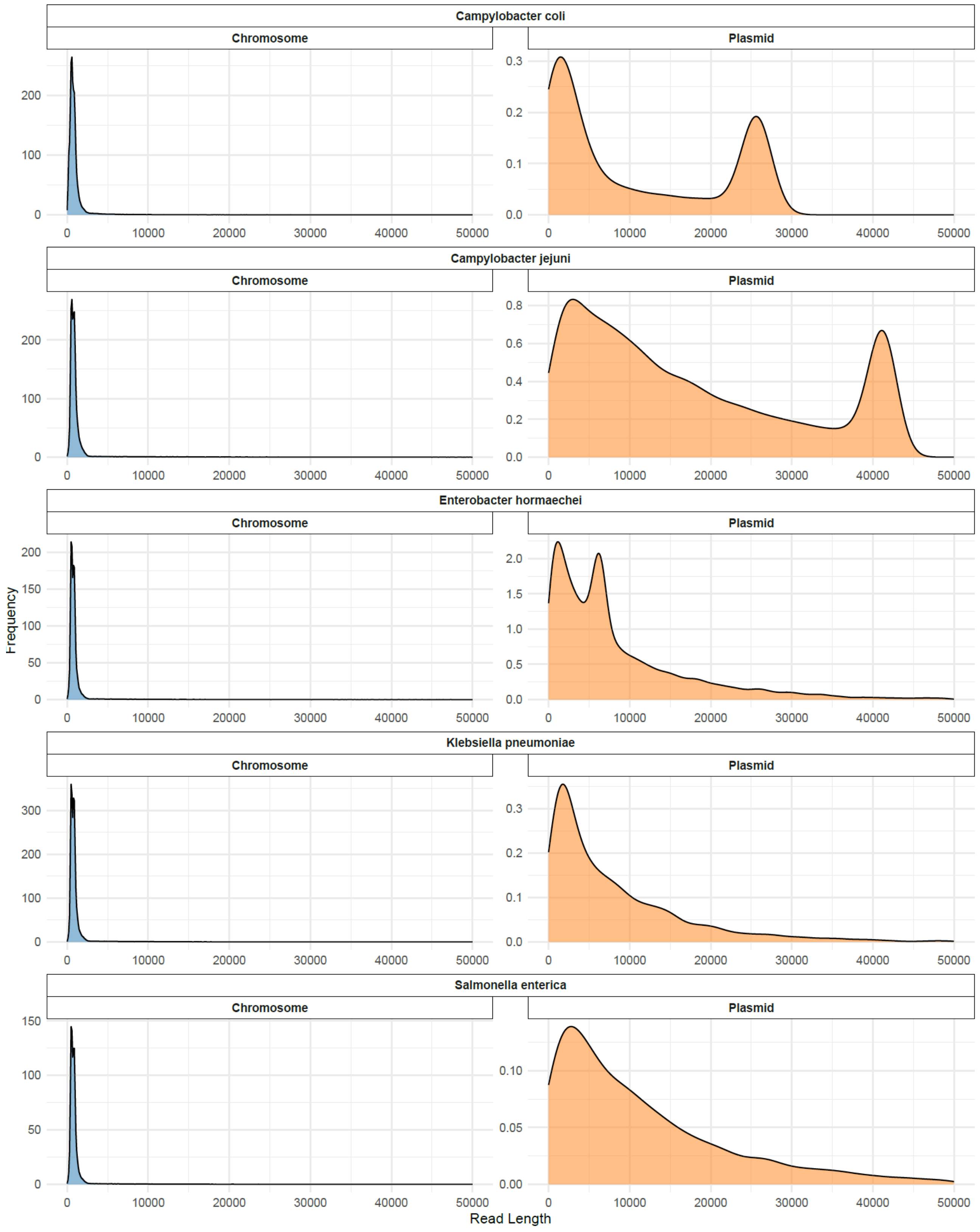

Supplementary Figure S4. Read length histograms of adaptive sampling regions from flow cells ReadBouncer1 and ReadBouncer2. Separate read length histograms of chromosomal and plasmid reads are shown for each of the five bacterial isolates. Chromosomal read lengths are consistently shorter than plasmid read lengths due to rejection of chromosomal reads.

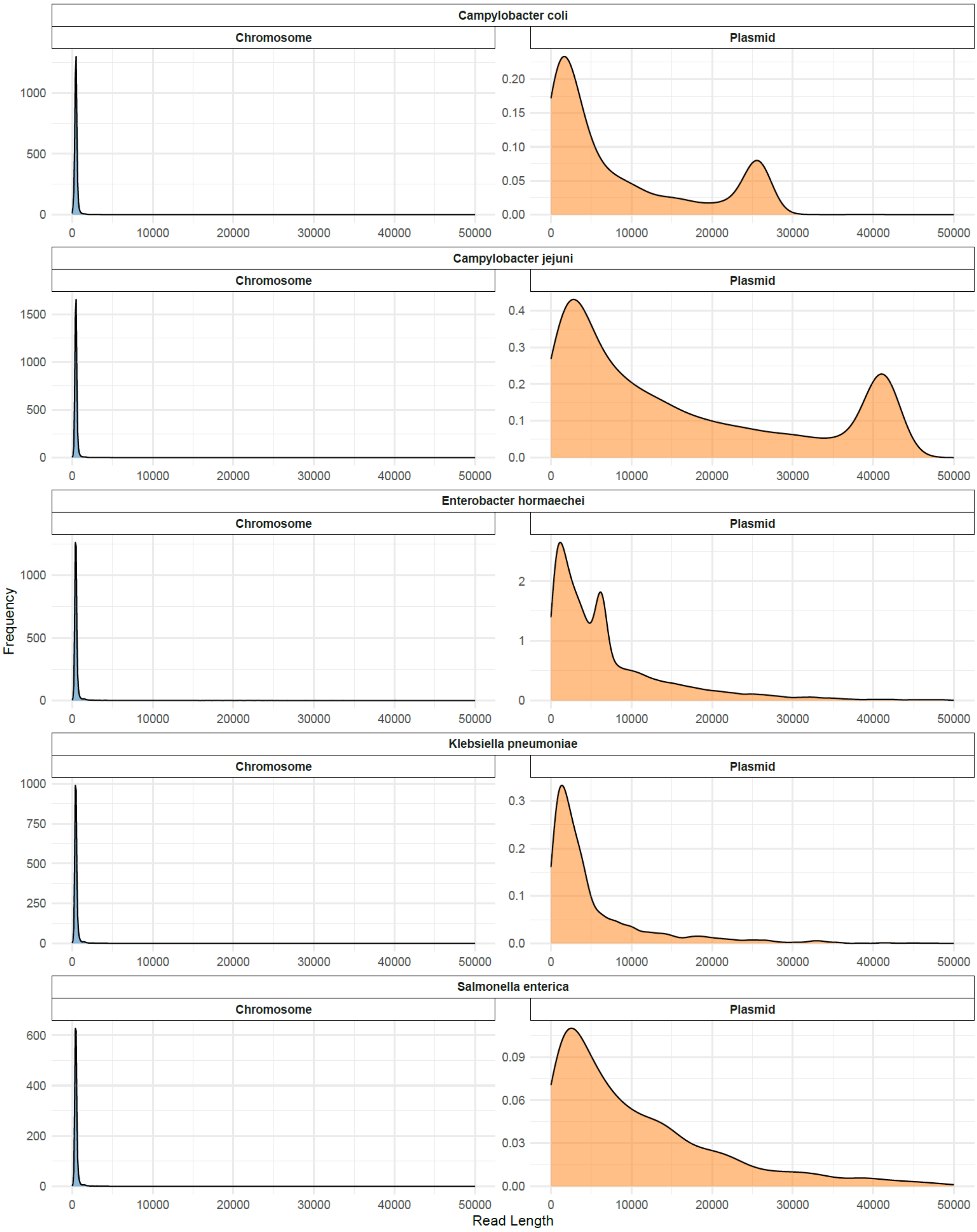

Supplementary Figure S5. Read length histograms of adaptive sampling regions from flow cells MinKNOW1 and MinKNOW2. Separate read length histograms of chromosomal and plasmid reads are shown for each of the five bacterial isolates. Chromosomal read lengths are consistently shorter than plasmid read lengths due to rejection of chromosomal reads.

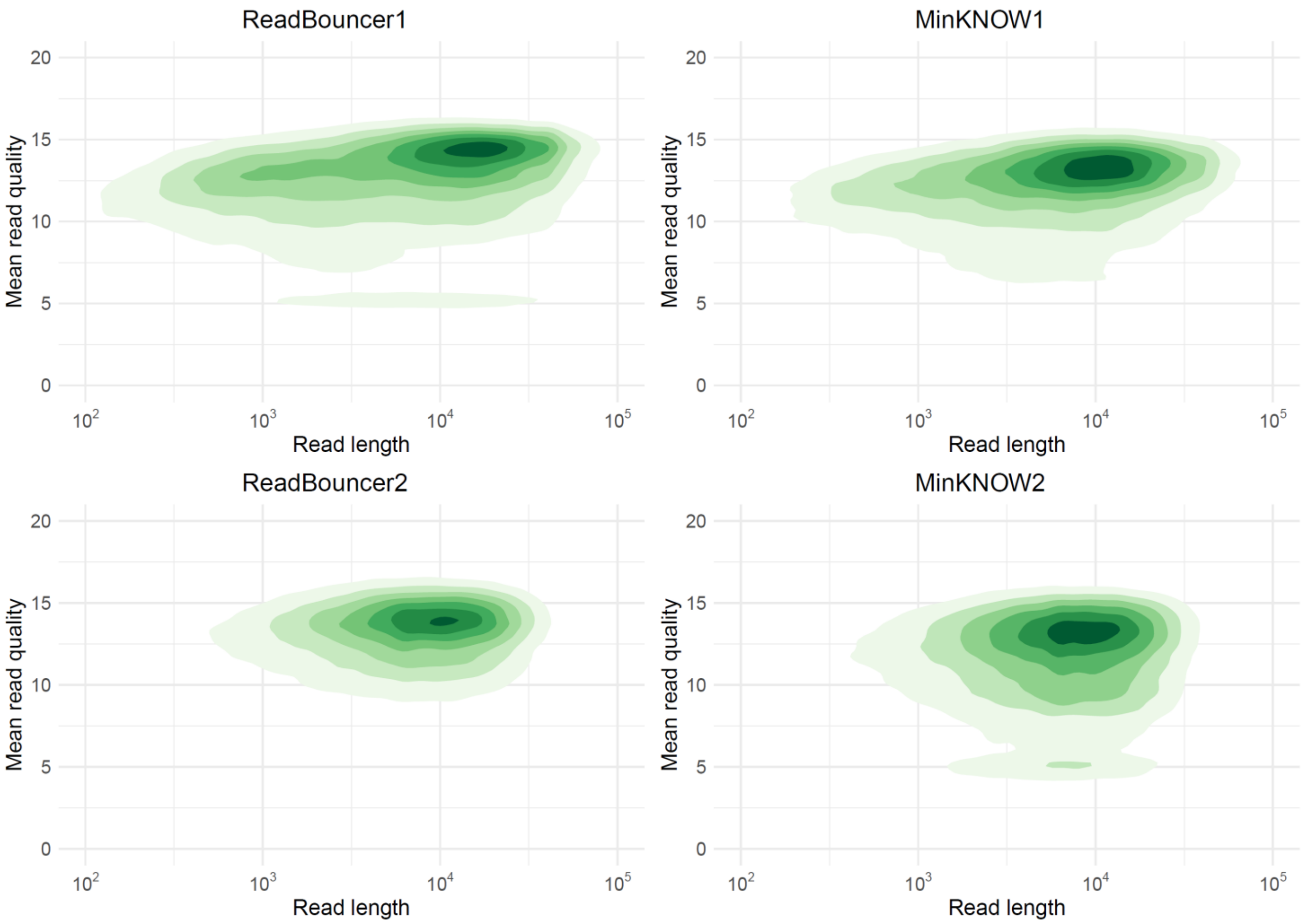

Supplementary Figure S6. Contour plots of read lengths (log scale) against mean read quality for control regions of the four sequencing runs. Darker regions indicate a higher proportion of reads that fall into that slice. For example, ReadBouncer1 and MinKNOW1 have a higher proportion of reads with length above 10,000 base pairs than for ReadBouncer2 and MinKNOW2.

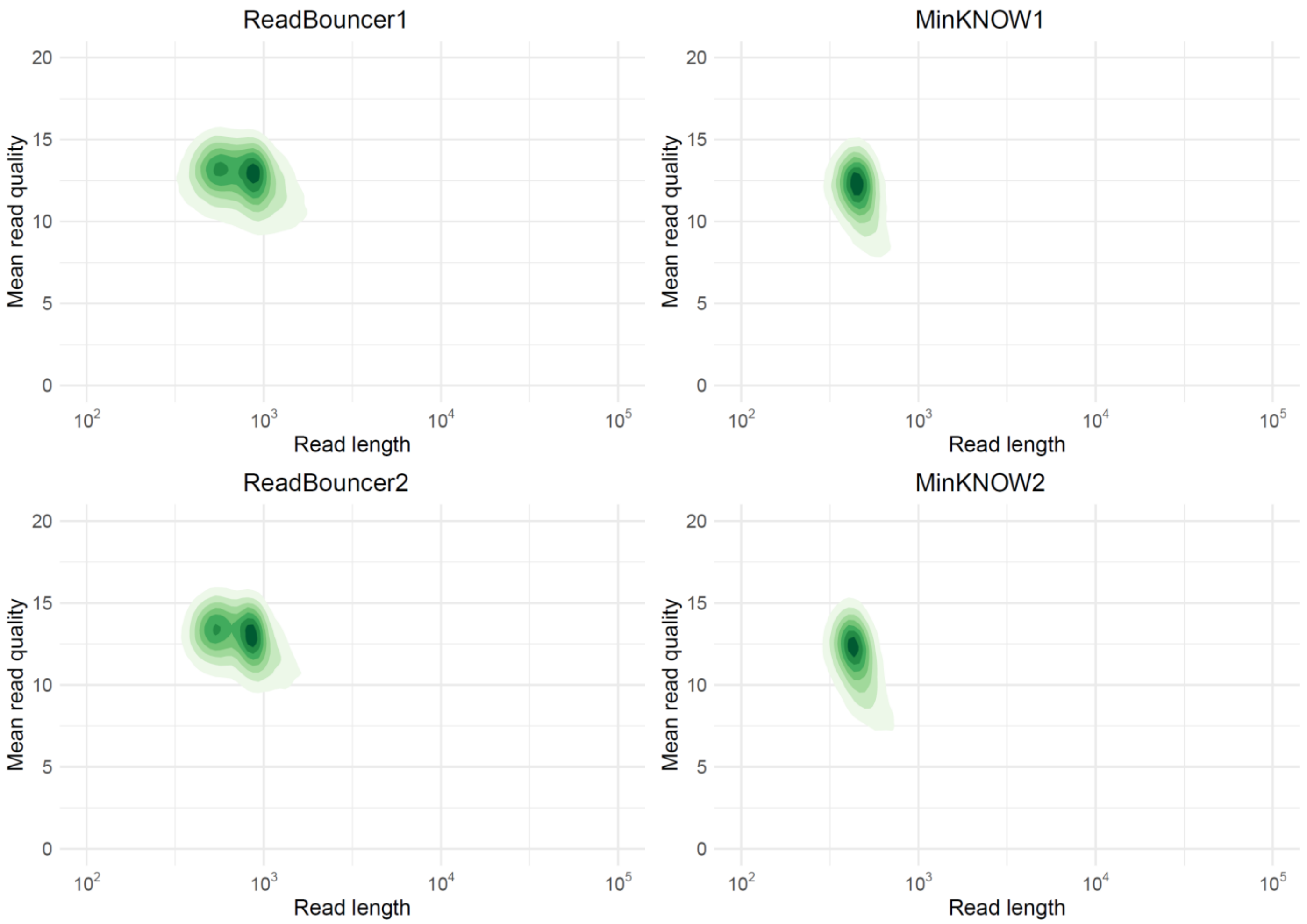

Supplementary Figure S7. Contour plots of read lengths (log scale) against mean read quality for adaptive sampling regions of the four sequencing runs. Darker regions indicate a higher proportion of reads that fall into that slice. For example, most reads from sequencing runs MinKNOW1 and MinKNOW2 have read lengths of about 650 base pairs and a Phred quality value of around 12.

(a)

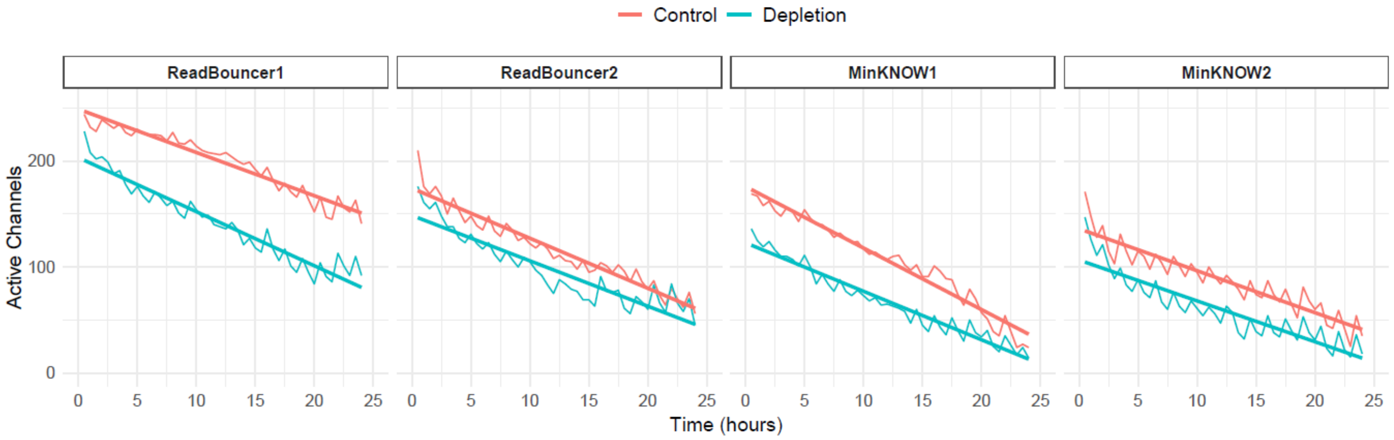

(b)

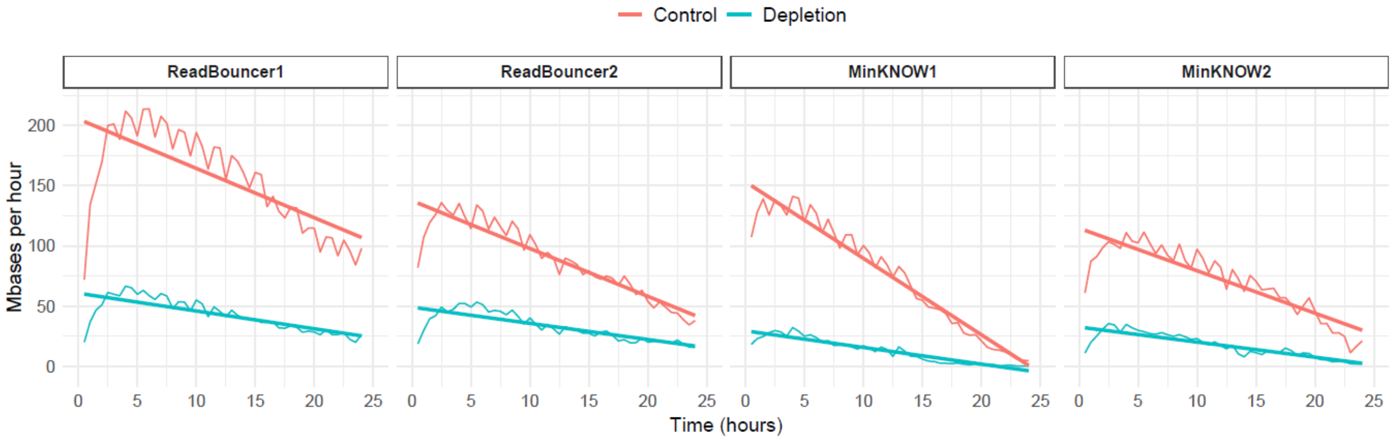

Supplementary Figure S8. Comparison of active channels and yield between control and adaptive sampling (depletion) regions in all four experiments. (a) Plots showing how the number of active channels varies with time in adaptive sampling (depletion) and control regions. There are more active sequencing channels in control regions on all four flow cells, but the there is no difference in deterioration of active sequencing channels between both regions. **(b)** Hourly yields from depleted channels vs. control channels. Usage of adaptive sampling results in lower overall sequencing yield compared to normal sequencing, but yield per hour decreases faster in the control regions.

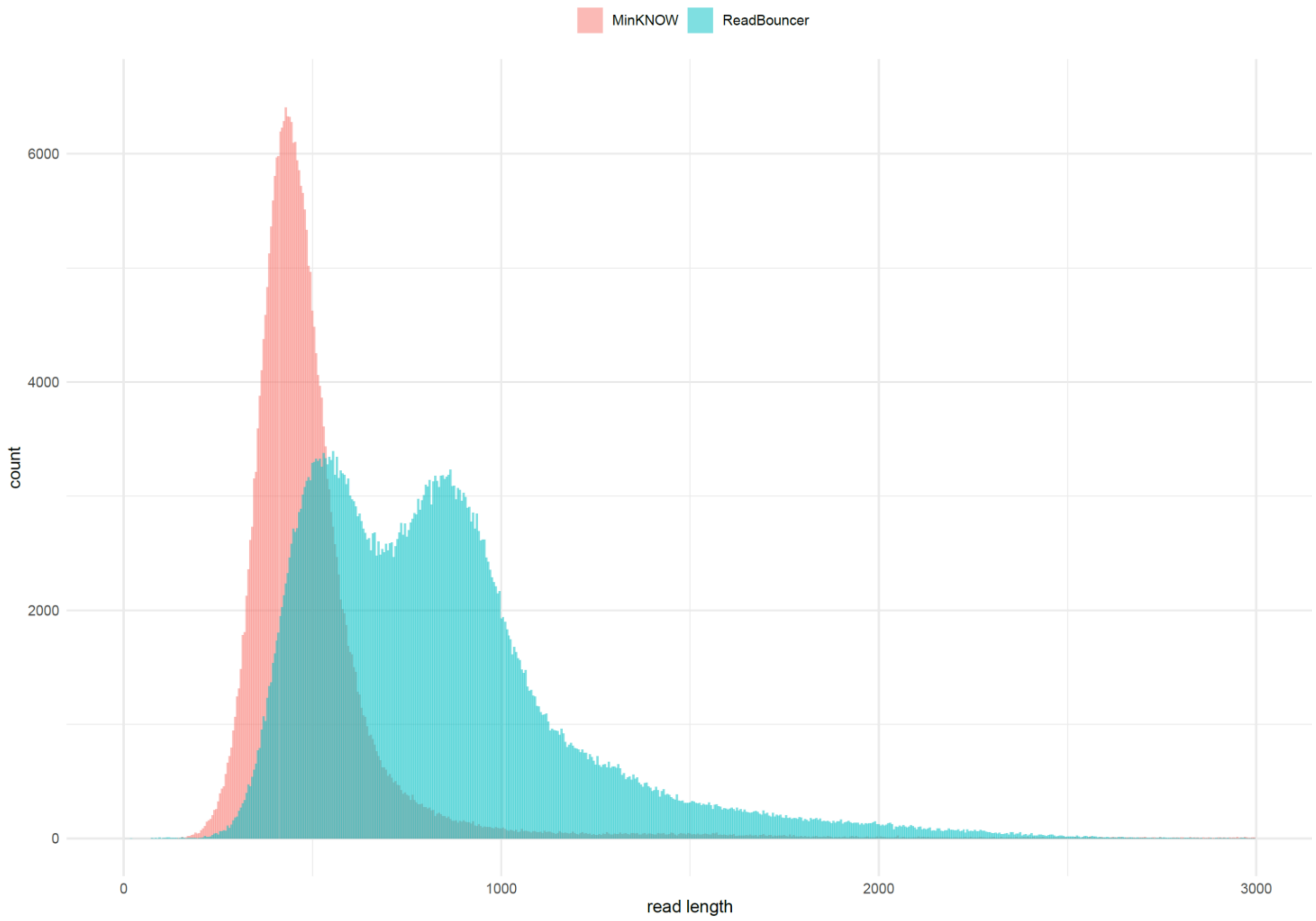

Supplementary Figure S9. Read length histogram of rejected reads by MinKNOW and ReadBouncer. Adaptive sampling with MinKNOW leads to rejected read lengths of about 450 to 500 nucleotides while many rejected reads are even longer than 1,000 nucleotides when using ReadBouncer
